# PepSeq as a highly multiplexed platform for melioidosis antigen discovery and vaccine development

**DOI:** 10.3389/fimmu.2025.1605758

**Published:** 2025-07-03

**Authors:** Evan A. Elko, Charles H. D. Williamson, Heather R. Green, Marcellene A. Gates-Hollingsworth, Georgia A. Nelson, Sujata G. Pandit, Heather L. Mead, Christopher Allender, Celeste Woerle, Mark Mayo, Bart J. Currie, David P. AuCoin, John A. Altin, Paul Keim, Erik W. Settles, Jason T. Ladner

**Affiliations:** ^1^ The Pathogen and Microbiome Institute, Northern Arizona University, Flagstaff, AZ, United States; ^2^ Department of Microbiology and Immunology, University of Nevada, Reno School of Medicine, Reno, NV, United States; ^3^ The Translational Genomics Research Institute, Flagstaff, AZ, United States; ^4^ Global and Tropical Health Division, Menzies School of Health Research, Charles Darwin University, Darwin, NT, Australia; ^5^ Infectious Diseases Department and Northern Territory Medical Program, Royal Darwin Hospital, Darwin, NT, Australia; ^6^ Department of Biological Sciences, Northern Arizona University, Flagstaff, AZ, United States

**Keywords:** PepSeq, highly multiplexed serology, melioidosis, *Burkholderia pseudomallei*, vaccine, epitope, GroEL

## Abstract

**Introduction:**

Vaccination aims to prevent or mitigate disease by priming the immune system prior to infection. While historical vaccine development relied mostly on trial-and-error, modern approaches have become more directed. By leveraging our growing understanding of pathogen biology and immune correlates of protection, we can design vaccines in ways that promote protective responses. However, the complexity of many pathogens (e.g., bacteria and fungi), as well as our immune responses against them, continue to present important challenges for vaccine development.

**Aim:**

Here, we demonstrate the utility of the PepSeq platform for highly multiplexed serology to both broadly and finely characterize antibody responses against complex pathogens, using the bacterium, *Burkholderia pseudomallei*, as a case study.

**Methods:**

We designed and synthesized three diverse pools of DNA-barcoded peptides (i.e., PepSeq libraries) and used them to characterize antibodies against a variety of *B. pseudomallei* proteins.

**Results:**

Epitope-resolved antibody binding profiles were generated for 85 individuals with culture-confirmed melioidosis, 89 US blood bank controls, and 6 monoclonal antibodies. Using these data, we identify novel B cell antigens/epitopes and finely characterize the epitopes of three monoclonal antibodies against the *B. pseudomallei* GroEL protein.

**Conclusion:**

Highly multiplexed serology platforms, like PepSeq, enable more comprehensive characterization of antibodies, both polyclonal and monoclonal, which can aid in the development of vaccines, diagnostics and therapeutics, even for pathogens with large, complex genomes.

## 1 Introduction

The goal of vaccines is to prevent or reduce disease through the stimulation of the immune system such that specific responses are primed before an infectious agent is encountered. While vaccination has had a long successful history that predates detailed knowledge of the immune system, these trial-and-error successes mask many attempts that failed to protect (1, 2). In contrast, modern vaccine development leverages our more detailed understanding of the mediators and/or correlates of protective immune responses to natural infection. In particular, the precise pathogen antigens that stimulate protection during an infection can be used in vaccines to similarly protect naïve hosts without encountering the actual infectious agent. But it is not always simple, as immune responses are complex, involving both cellular and humoral components, and pathogens always display multiple, frequently many and sometimes diverse, antigens that stimulate responses. Precisely identifying which of the responses are protective, which are not, and the particular antigens that invoke protection is difficult; comprehensive characterization of the complex responses is the logical starting point and is increasingly feasible with highly multiplexed assay technologies.

Characterizing immune complexity requires multiple approaches with highly multiplexed capacity to provide sufficiently broad bandwidth to comprehensively cover antigenic determinants and their variability within pathogens. The “Omic sciences” have significantly increased our bandwidth for meaningfully monitoring both pathogen and host diversity (3). Genomics was perhaps first in this regard, but proteomics and metabolomics are now affordable and also generate highly multiplexed data. Similar approaches for characterizing complex immune responses are essential and many have been developed that capitalize upon the Omic science successes. This includes both cellular and humoral responses where genomic technologies are characterizing T and B cell changes at the cellular level. The analysis of antibody responses to infectious agents have been regularly performed since the advent of serology by Karl Landsteiner (4) and Paul Ehrlich (5) in the late 19^th^ century. While the analysis and understanding of humoral responses has advanced greatly in the last 100 years, highly multiplexed analysis of antibodies is a recent innovation. In this paper, we present methodologies and data for high resolution and highly multiplexed approaches for understanding antibody complexity using the PepSeq platform.

PepSeq utilizes diverse libraries of roughly epitope sized peptides that are each uniquely barcoded with a DNA tag. These libraries can easily be designed from proteome predictions of genomic sequences (6, 7) and they can be used to interrogate biological fluids for antibody binding to hundreds of thousands of antigens/epitopes. PepSeq’s multiplex capacity is limited primarily by oligonucleotide synthesis capacities, a barrier that has been greatly reduced with advancements in programmable nucleic acid synthesis technologies. It is now possible to design and economically produce a 244,000 peptide library in only a few weeks (7). This allows for coverage of entire pathogen proteomes in a single assay and with epitope-level resolution (8, 9), even for the larger proteomes of cellular pathogens, like bacteria and fungi.

In prior work, we have developed and used a variety of PepSeq libraries representing the human virome. Among other applications, we have used these libraries to (i) profile infection histories (8, 10), (ii) identify/characterize novel epitopes (including targets of broadly-neutralizing antibodies) (9–11), (iii) track the dynamics of vaccine responses (11), and (iv) classify infection or seropositivity status with high accuracy (9, 12). The goal of the present study is to demonstrate the utility of PepSeq for both broadly and finely characterizing antibody responses to more complex bacterial pathogens, using *Burkholderia pseudomallei* as a case study.

## 2 Materials and methods

### 2.1 Design and synthesis of PepSeq libraries

**Figure 1:**
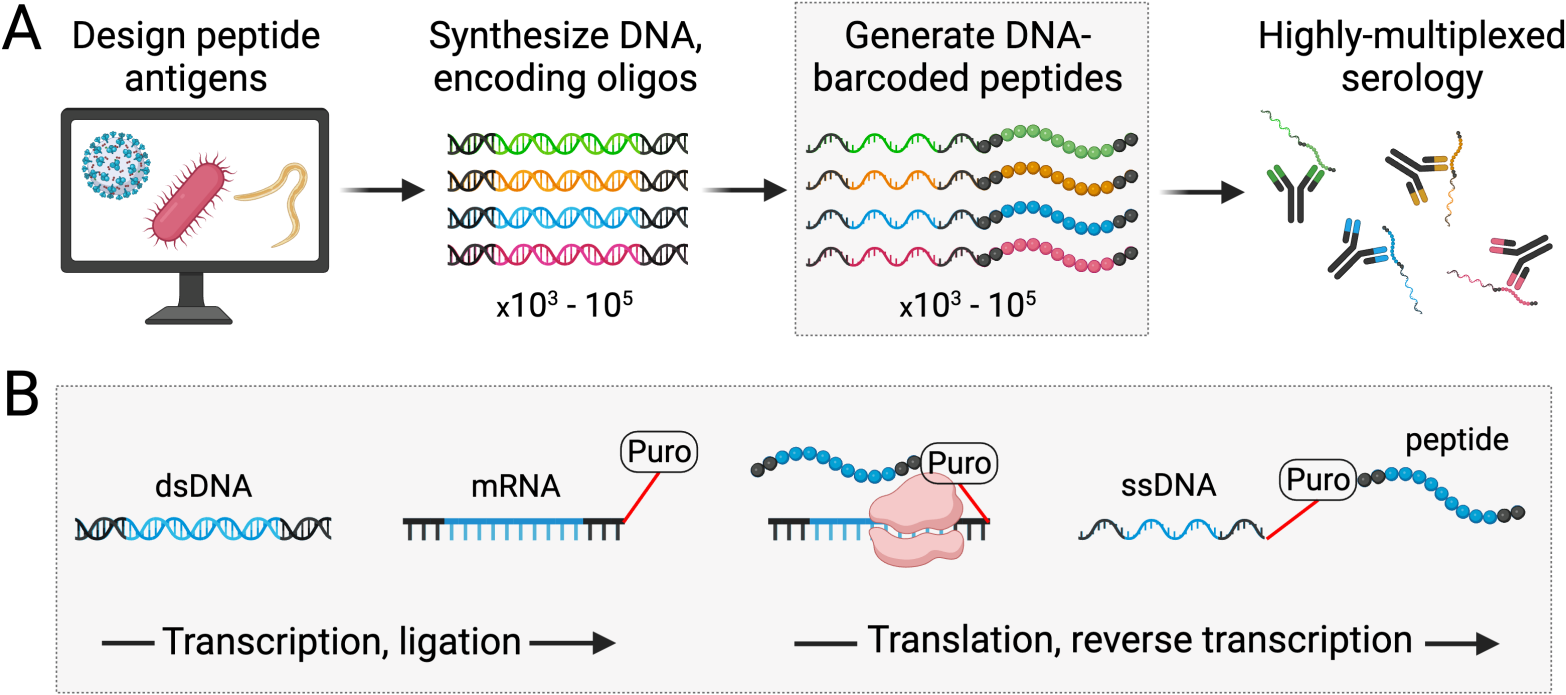
Generation of PepSeq probes for use in highly multiplexed serology. PepSeq probes are DNA-barcoded peptides with sequences that are fully defined and customizable. The first step in generating PepSeq probes is the design and synthesis of DNA oligonucleotides encoding peptides of interest **(A)**. Through a series of fully *in vitro* transcription, ligation and translation reactions, these DNA oligonucleotides are then converted into peptides covalently linked to their encoding cDNA **(B)**. Critically, the puromycin-mediated intermolecular coupling of peptide to mRNA allows diverse pools of oligonucleotides to be processed, in bulk, producing equally diverse libraries of PepSeq probes **(A)**. Black regions of the nucleic acids and peptides represent conserved adapters present in all PepSeq probes, while the multicolored **(A)** or blue **(B)** segments correspond to each specific antigen [see Henson et al. (7) for details]. Created with BioRender.com.

PepSeq libraries are diverse pools of DNA-barcoded peptides (i.e., “probes”) that can be synthesized, en masse, starting with DNA oligonucleotides that code for the peptide antigens of interest (**Figure 1**). Commercially sourced pools of fully specified DNA oligonucleotides are used as the starting material for synthesizing PepSeq probes, and we have successfully generated PepSeq libraries with as many as 244,000 peptide antigens and with individual peptides up to 64 amino acids in length. The PeqSeq library synthesis protocol has already been described, in detail (7), and is summarized in **Figure 1**. In brief, the DNA oligonucleotide templates are *in vitro* transcribed using T7 RNA polymerase to generate transcripts suitable for *in vitro* translation. A puromycin linker is ligated to the RNA, which terminates the translation and covalently links the RNA to its specific peptide. Finally, the RNA is reverse transcribed to form ssDNA and the RNA is digested. The cDNA copy of the transcript serves as a unique tag for monitoring the relative abundance of each peptide via high-throughput sequencing, and it is this link between peptide antigens and DNA tags that enable the use of PepSeq libraries for highly multiplexed serology.

Here, we illustrate the utility of the PepSeq platform for highly multiplexed serology to characterize antibodies elicited by *Burkholderia pseudomallei*, across the full proteome and with epitope-level resolution. To explore antibody reactivity patterns against *B. pseudomallei* proteins, we designed and synthesized three PepSeq libraries, including two different versions (BKP1 and BKP2) broadly targeting the *B. pseudomallei* proteome and one focused on an in-depth characterization of specific antibody epitopes (MUT) (see below for details). We report on the use of these libraries to 1) identify novel protein antigens and B cell epitopes using proteome-wide antibody surveys and 2) identify and finely characterize the epitopes of monoclonal antibodies.

### 2.2 PepSeq antibody binding assays

PepSeq antibody binding assays were conducted as previously described (7). Briefly, each assay involves the incubation of antibodies (e.g., polyclonal populations within serum, purified monoclonal antibodies) with a diverse pool of PepSeq probes. Immunoglobulin (Ig) is then precipitated using capture proteins bound to magnetic beads, non-binding PepSeq probes are washed away, and the relative abundance of each probe is quantified using PCR and high-throughput sequencing of the DNA portion of the molecules. Here, we used Streptococcal protein G as our capture protein, which allowed us to examine the total IgG response. However, the same methodology can be adapted to other isotypes simply by modifying the bead-bound capture protein. Following PCR, a standard bead cleanup was performed, and products were individually quantified (Quant-It, Thermo Fisher), pooled, re-quantified (KAPA Library Quantification Kit, Roche) and sequenced on an Illumina instrument.

PepSeq sequencing data was processed and analyzed as previously described (7) using PepSIRF v1.6.0 (13) as well as associated Qiime2 plugins (14) and custom python scripts (https://github.com/LadnerLab/PepSIRF/tree/master/extensions). First, the reads were demultiplexed and assigned to peptides using the PepSIRF *demux* module, allowing for one mismatch in each index sequence and three mismatches in the variable DNA tag region. The PepSIRF *norm* module was then used to normalize counts to reads per million (RPM). RPM normalized reads from 2–8 buffer-only control samples were subsequently used to create bins for Z score calculation using the PepSIRF *bin* module. To normalize for different starting peptide abundances within each bin, reads were further normalized by subtracting the average RPM from the buffer only controls (–diff option in *norm* module). Z scores were calculated using the PepSIRF *zscore* module using the 95% highest density interval within each bin.

### 2.3 Sera and monoclonal antibodies used in this work

Serial venous whole blood was collected from 85 culture-confirmed patients with melioidosis from the Darwin Prospective Melioidosis Study (15) and the associated blood collection protocol was previously described (16). Enrollment and sample collection was approved by the Human Research Ethics Committee of the Northern Territory Department of Health and the Menzies School of Health Research (HREC 02/38 and HREC 2014-2037).

Non-endemic healthy control samples (n=89) were collected by Creative Testing Solutions from California as previously described (16). These samples were deemed exempt by the Northern Arizona University Institutional Review Board (IRB). These samples were selected for comparison since they are from individuals who are unlikely to have been exposed to *B. pseudomallei*. In addition, local endemic controls were not used since *B. pseudomallei* exposure history is typically not known with certainty. Individuals living in endemic regions can have low grade disease that does not require intervention.

To generate hybridomas and monoclonal antibodies (mAbs) against *B. pseudomallei* K96423 GroEL, mice were vaccinated using purified GroEL. The use of laboratory animals in this study was approved by the University of Nevada, Reno Institutional Animal Care and Use Committee (protocol number 00024) and performed in conjunction with the Office of Lab Animal Medicine, which adheres to the National Institutes of Health Office of Laboratory Animal Welfare (OLAW) policies and laws (assurance number A3500-01). GroEL was selected as a target because it is known to be highly immunogenic across multiple host species and in response to multiple bacterial pathogens (17–23).

Expression and purification of N-terminally His6-tagged GroEL was previously described (16). Briefly, GroEL1 (BPSL2697) was cloned into an *Escherichia coli* expression vector and expressed in *E. coli*. Tagged protein was purified using nickel affinity chromatography (HisPrep FF 16/10) and quantified by Bradford assay. Purity was estimated by SDS-PAGE and Sypro Ruby stain. BALB/C mice were vaccinated with recombinant GroEL and Freund’s adjuvant. Hybridomas were generated as previously described (24). Hybridomas that secrete anti-GroEL antibodies were identified using a GroEL ELISA. Six monoclonal antibody (mAb) clones were identified, expanded, and the resulting antibodies were purified by Protein A affinity chromatography. All of the resulting mAbs were shown to bind to full-length, native GroEL protein. The isoptypes of these mAbs were IgG1 (15E2, 18E1, 18E7), IgG2a (7D10, 8E4) and IgG2b (10A4).

### 2.4 PepSeq library to identify novel B cell antigens

**Figure 2:**
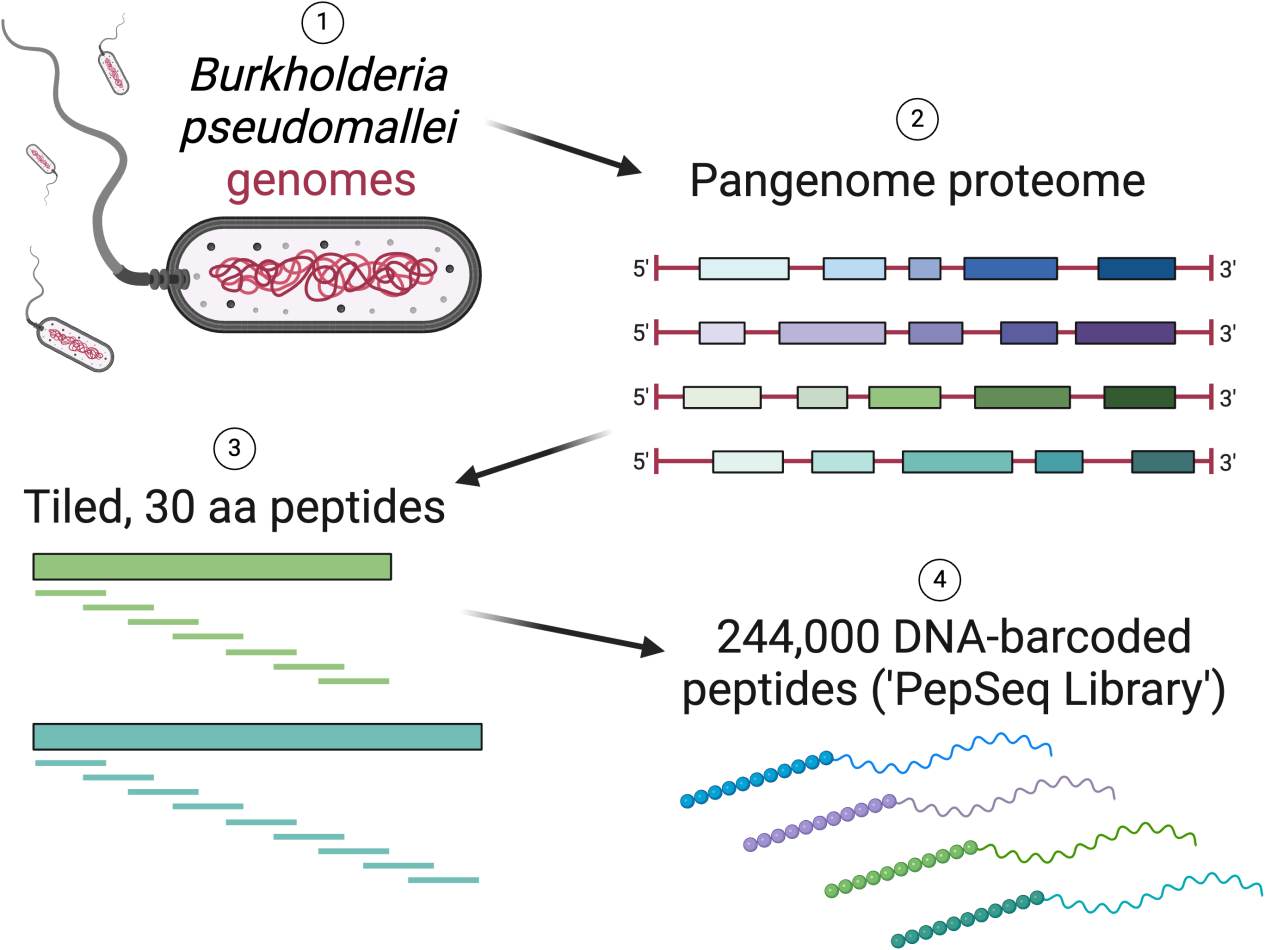
Overview of the design of a PepSeq library (BKP1) with peptides tiled across the *Burkholderia pseudomallei* “pan-proteome”. Created with BioRender.com.

The BKP1 library contains 244,000 target peptides, each of which is 30 amino acids in length (**Figure 2**, **Supplementary Table 1**). These peptides were designed to broadly cover the antigenic diversity present across the “pan proteome” of *B. pseudomallei*. Specifically, we designed peptides from 12,174 protein clusters (see **Supplementary Methods** for details on the selection of protein sequences and clusters) for an average of ~20 peptides per cluster. Peptides were designed using our previously described sliding window + set cover algorithm (7) (https://github.com/LadnerLab/Library-Design/tree/master/SW_SC/python). In brief, this involved the selection of one representative sequence from each cluster, across which peptides were tiled with a step size of 22 amino acids, resulting in an 8 amino acid overlap between adjacent peptides. A set cover algorithm was then used to select additional peptides to cover variability among sequences within the same cluster. Additional peptides were added to the design until a specified percentage of unique cluster 9mers had been covered, with the threshold for each cluster set dynamically based on cluster redundancy (as implemented in https://github.com/LadnerLab/Library-Design/blob/master/extensions/dynamicThresholds.py). Finally, we removed low complexity peptides, which are less likely to provide antibody signatures specific for *B. pseudomallei* (for details see **Supplementary Methods**) and redundant peptides (i.e., if all 6mers from one peptide were contained in other design peptides) (https://github.com/LadnerLab/Library-Design/blob/master/extensions/kmerRedundancy.py). In addition to the *B. pseudomallei* peptides, as positive controls, we included 380 virus derived peptides known to be commonly targeted by human IgG antibodies (9, 12).

All serum samples characterized with our BKP1 PepSeq library were assayed in duplicate. To ensure data quality, only samples which met our raw read count threshold (>244,000 raw sequence reads in each replicate) and showed strong correlation of enriched peptides (Z score > 6) between replicates (Z score Pearson’s correlation >0.6) were included in downstream analyses. We obtained an average of 1.1M Illumina sequencing reads per replicate, which equates to an average of 4.5 reads per unique BKP1 peptide per replicate.

To identify B cell antigens targeted in response to *B. pseudomallei* infection, we compared antibody reactivity profiles for 85 Australian melioidosis patients and 89 USA blood bank controls. To identify peptides with diagnostic potential, we focused on the subset that showed reactivity in at least ~5% of the melioidosis patient samples (Z score >6 in ≥4 samples, n=4,435 peptides). For each of these peptides, a significance threshold was calculated as three standard deviations above the mean Z score of the negative control samples. These peptide level thresholds were used to count the number of positive and negative samples in each cohort, and then, for each peptide, we calculated the 1) positive predictive value [true positives/(true positives + false positives)] and 2) sensitivity [true positives/(true positives + false negatives)]. A p-value was calculated for each peptide using a two-sided Fisher’s Exact test with a Benjamini-Hochberg multiple test correction. Peptides of interest were identified as those with a corrected p-value ≤ 0.05. To assess our false discovery rate, we followed an identical procedure to also identify peptides with higher reactivity in 1) our control cohort compared to our melioidosis cohort and 2) datasets with randomly permuted cohort assignments (n=1000).

To assess the diagnostic performance of the identified peptides within this cohort, both individually and when summed together, we generated receiver operating characteristic (ROC) curves with Z score thresholds ranging from -2 to 228. To determine the smallest subset of peptides that yielded the largest AUC, we applied both forward and backward selection models, requiring a minimum increase in AUC of 0.001 when adding (or subtracting) a peptide. At each step, the peptide that resulted in the largest AUC increase was selected for inclusion (or exclusion).

To estimate the evolutionary conservation of proteins of interest (i.e., proteins from which identified peptides originated) across *Burkholderia* strains and species, the predicted proteomes for 1024 genomes representing 26 *Burkholderia* species were screened for 65 proteins of interest (**Supplementary Table 2**) with LS-BSR (25) using the BLASTP v2.9.0+ (26) alignment option. A predicted protein was considered conserved for a screened proteome at a BSR value threshold of >0.7. Some species were represented by only one predicted proteome.

### 2.5 PepSeq libraries to identify and finely characterize monoclonal antibody epitopes

A high-density *B. pseudomallei* peptide library (BKP2) was generated to identify and refine monoclonal antibody epitopes. This library was used to screen our six, previously generated anti-GroEL mAbs. In full, the BKP2 library contained 30,972 probes (each 19 aa in length; **Supplementary Table 3**) and was designed to cover 192 *B. pseudomallei* K96243 predicted coding regions, along with a variety of positive and negative control peptides (559 control peptides in total). Both GroEL1 (BPSL2697) and GroEL2 (BPSS0477) were included as targets. The *B. pseudomallei* peptides were tiled across each full-length protein, starting every other amino acid (i.e., a step size of 2 aa). In total, this library contained 264 probes designed to cover GroEL1 and another 264 designed to cover GroEL2, though in some instances, due to shared homology, probes designed to cover GroEL1 encoded peptides identical to those designed to cover GroEL2. In cases where our library included multiple nucleotide encodings for the same peptide, we separately calculated Z scores for each encoding but report only the maximum Z score per peptide within relevant figures.

In part, the power of the PepSeq platform comes from rapid capacity to redesign libraries based upon experimental results, to test and refine hypotheses. Initial experiments, for example can be used to identify likely epitope peptides with subsequent libraries testing alterations of those sequences to refine our understanding of antibody binding footprints. Not only can the epitope borders be defined, but internal amino acid interactions can be examined through exhaustive substitution at individual residues. Using the BKP2 library, we identified two regions of GroEL1/2 targeted by a subset of our anti-GroEL mAbs (see section 3.2). Using these “minimum epitopes” as a starting place, we designed a new library (MUT) that contained a “parent” 30 aa long peptide for each epitope (designed from GroEL1 and with the minimum epitope in the center), as well as 620 peptide variants for each parent, which were used to finely characterize mAb binding to these epitopes (**Supplementary Table 4**). The sequences for the parent GroEL1 epitope peptides are ELDVVEGMQFDRGYLSPYFINNPDKQVAVL (for mAbs 8E4 and 18E7) and RVKQIRTQIEEATSDYDREKLQERVAKLAG (for mAb 7D10).

To further characterize the minimum binding footprint of each mAb, 50 “shift” peptide variants were included for each parent peptide. These variants involved the sequential removal of parent amino acid residues (1–25 amino acids per peptide) from both the N and C termini (individually) with replacement, to the opposite terminus, of an equal number of residues randomly selected from a subset of amino acids: alanine, glycine, serine and threonine. These peptides allowed us to isolate differently sized stretches of sequence, from both termini of the parent peptide, without the potentially confounding addition of adjacent portions of the GroEL protein. To pinpoint specific amino acids that are critical for mAb binding, we also designed 570 “substitution” peptide variants for each parent peptide. These variants were generated by individually substituting the wild-type amino acid at each of the 30 positions of the parent peptide to each of the alternative 19 amino acids, with each variant differing by a single amino acid from the relevant parent. This design allowed us to move beyond a traditional alanine scan approach for mapping epitopes because we can simultaneously (and cost-effectively) evaluate the impact of any substitution at all residues.

Given the large number of potential ligands that can be included in a PepSeq library, many different epitopes can be examined in a single experiment. In total, the MUT library included peptides designed to cover 615 antibody epitopes (see **Supplementary Methods** for details). Non-GroEL peptides are used here as negative controls, to assess the specificity of our mAbs.

### 2.6 Antibody affinity measurements by surface plasmon resonance

Surface plasmon resonance (SPR) was used to assess functional affinities of three mAbs (8E4, 18E7 and 7D10) using recombinant GroEL protein and of two of these mAbs (8E4 and 18E7) using a GroEL peptide (EGMQFDRGYLSPYFINNPD). Using a Biacore X100 instrument (Cytiva), recombinant protein was immobilized via amine chemistry to a CM5 sensor chip. The biotinylated GroEL peptide was immobilized to a sensor chip functionalized with streptavidin. Antibody binding to each immobilized ligand was measured by injecting mAb at multiple concentrations over the sensor surface. Assays were performed using 0.1 M HEPES, 1.5 M NaCl, 0.03 M EDTA and 0.5% v/v Surfactant P20 (Cytiva) as a running buffer and diluent. At least five antibody concentrations were used for analysis of binding kinetics and affinity. The dissociation constant (K_D_) was calculated using the steady-state equilibrium model and binding kinetics (k_a_ and k_d_) were evaluated using the bivalent model in BIA evaluation software.

## 3 Results

### 3.1 Melioidosis sera surveyed for antibodies against the *B. pseudomallei* proteome

Using a PepSeq library that covered the entire *B. pseudomallei* proteome (BKP1), we surveyed 85 Australian melioidosis and 89 USA blood bank control sera for antibody reactivity against *B. pseudomallei* proteins. Consistent with expectations, we observed strong antibody reactivity against a subset of our virus-derived, control peptides in all samples from both cohorts (11–121 out of 380 peptides with Z score ≥6 in both replicates). However, on average, we observed a larger number of reactive viral peptides in our negative control cohort (USA blood bank samples; avg. difference = 4.7, p=0.086, t-test) (**Supplementary Figure 1A**). Notably, this pattern was reversed when looking at the *B. pseudomallei* derived peptides (avg. difference = 84.2, p=0.0001, t-test) (**Supplementary Figure 1B**), consistent with the detection of specific antibody responses against *B. pseudomallei* in our melioidosis cohort.

The overall level of anti-*B. pseudomallei* reactivity varied considerably between samples, with 51-1,813 (median=215) peptides with Z scores ≥6 (in both replicates) in our melioidosis cohort. We also observed substantial variability in the specific proteins and peptides targeted by these antibody responses. Across the 85 samples from individuals with documented infections with *B. pseudomallei*, we observed a total of 21,262 enriched peptides derived from 7,996 different protein clusters. We also observed antibody reactivity against a considerable number of *B. pseudomallei* derived peptides within our negative control cohort [37-2,428 (median = 122) peptides with Z scores ≥6], even though these individuals were sampled in the USA, which is a non-endemic region for *B. pseudomallei*, and therefore it is unlikely that any of these individuals have had melioidosis. This result illustrates the importance of controlling for non-specific signals that likely result from antibodies that cross-recognize *B. pseudomallei* antigens, even though they were stimulated by other proteins (e.g., conserved domains in other bacterial pathogens).

**Figure 3:**
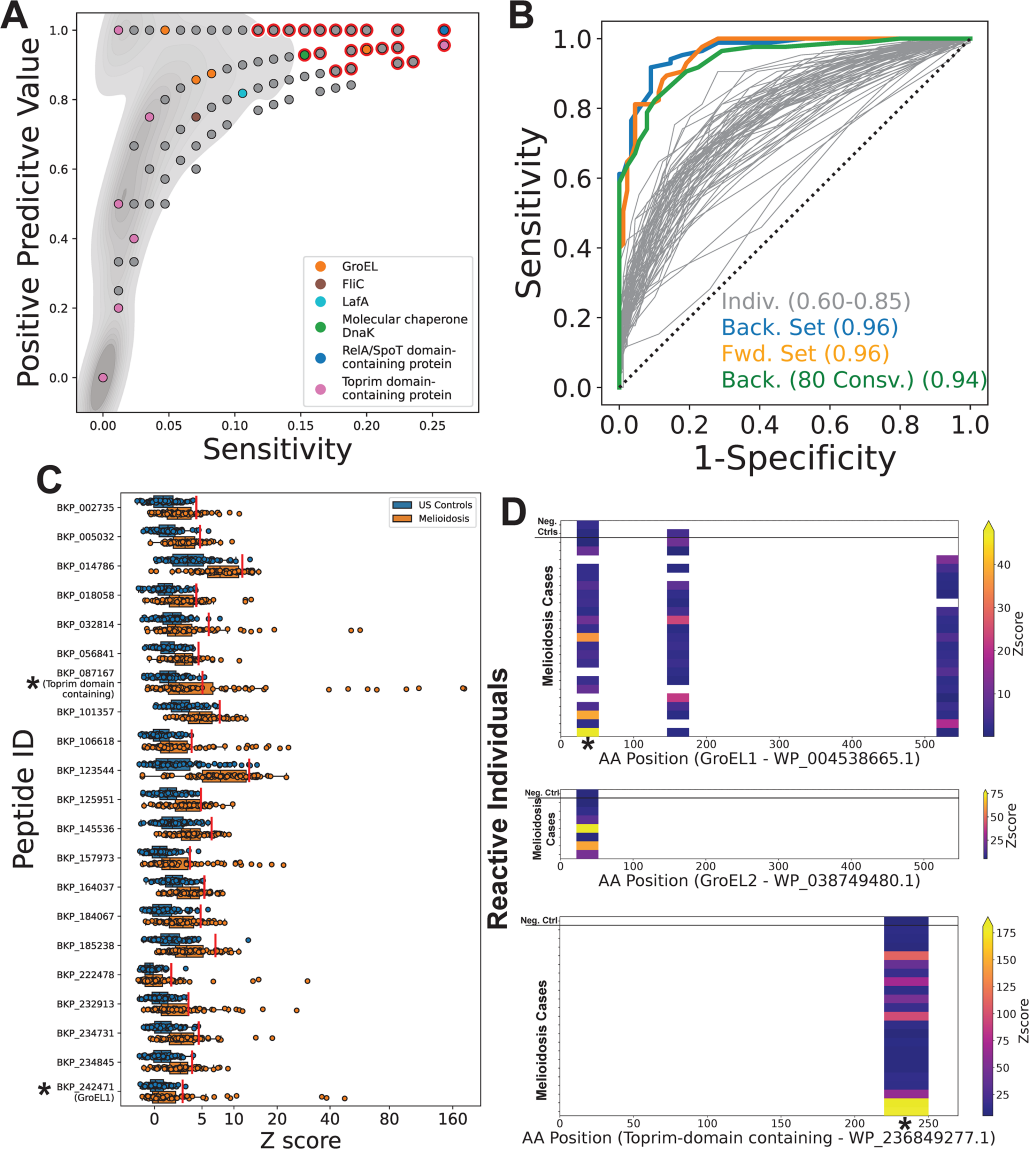
PepSeq identifies candidate diagnostic peptides from both known and novel protein immunogens. A PepSeq library (BKP1) covering the full pangenome proteome of *B. pseudomallei* was used to broadly assess anti-*B. pseudomallei* antibody reactivity in 85 Australian melioidosis patient sera and 89 control sera from a non-endemic, US population. **(A)** For a subset of 4,435 peptides recognized by IgG antibodies in ≥4 serum samples, the positive predictive value is plotted versus the sensitivity for our melioidosis cohort. Red outline indicates the positions of peptides that were significantly elevated in melioidosis samples (corrected p<0.05, two-sided Fisher’s Exact Test). Grey shading in background indicates the density of points. Six protein examples are labeled: GroEL=WP_038749480.1, WP_004522244.1, WP_038757344.1, WP_004538665.1; FliC= Q3JY46; LafA=WP_004523666.1; DnaK=WP_004533561.1, WP_004194034.1; RelA/SpoT=WP_004544909.1, Toprim domain-containing=WP_236849277.1. **(B)** ROC curves for the 67 peptides identified as significant after multiple test correction (grey lines). The set of peptides whose sum of Z scores resulted in the highest AUC was determined by both forward (orange line, n=12) and backward (blue line, n=10) selection. We similarly selected a maximally diagnostic set of peptides (n=7) while ensuring that all peptides were designed from proteins that have >80% sequence conservation across strains of *Burkholderia pseudomallei* (green line). **(C)** Antibody reactivity for 21 peptides identified as having the greatest diagnostic potential [i.e., present in ≥1 of the sets shown with colored lines in **(B)**] is shown for each serum specimen (n=174). X-axis indicates Z score of each peptide and the y-axis shows the peptide ID. Each circle represents an individual sample. The line within each box represents the median, while the lower and upper bounds of each box represent the first and third quartiles, respectively. The whiskers extend to points that lie within 1.5 interquartile ranges of the first and third quartiles. Red lines show the Z score threshold calculated for each peptide. Asterisks indicate peptides from proteins for which reactivity heatmaps are shown in panel **(D)**; the highlighted GroEL1 peptide is at amino acid (AA) positions 22-52. **(D)** Heatmaps showing the location of reactive peptides in the known antigens GroEL1 (top panel) and GroEL2 (middle panel), as well as a novel antigenic protein annotated as a Toprim domain containing protein (bottom panel). Each row is an individual serum specimen, and the x-axis shows the amino acid (AA) sequence position for the indicated protein. The color of the heatmap indicates the enrichment Z score. Asterisks indicate the peptides for which the Z scores are shown in panel **(C)**.

By comparing antibody reactivity profiles between our case and control cohorts, we identified 67 candidate diagnostic peptides for melioidosis (corrected p-value ≤ 0.05) (**Figure 3A**, red highlighted points). In contrast, none of our randomly permuted datasets (n=1000) contained peptides that met these same criteria. Therefore, we can be confident that most of these 67 peptides represent true biological differences between our cases and controls, rather than statistical artifacts. However, using identical thresholds, we also identified 18 *B. pseudomallei* derived peptides as candidate diagnostic peptides for our negative controls (i.e., peptides with consistently higher reactivity in our control cohort) (**Supplementary Figure 2**). This result suggests that a subset of our candidate diagnostic peptides for melioidosis (~27%) potentially reflect other differences in exposure histories between our Australian (melioidosis) and USA (control) cohorts, independent of *B. pseudomallei*.

Even within our subset of candidate diagnostic peptides for melioidosis, peptide-level sensitivity was relatively low (≤0.26, average = 0.18), reflecting highly variable responses across individuals. However, positive predictive value was high for many peptides, including several known immunogens (**Supplementary Figure 3**). For example, one peptide from the N terminal region of GroEL (18) had a PPV of 0.944 but was only targeted by IgG antibodies in 20% of our samples from individuals with documented *B. pseudomallei* infections. We also saw strong antibody reactivity against two *B. pseudomallei* Flagellin proteins (FliC/GenBank: ABA48561.1 and LafA/GenBank: WP_004523666.1) (18), though strong binding to the same FliC peptides were also observed in several negative controls (**Supplementary Figure 3**), indicating the potential for non-specific cross-reactivity, likely driven by sequence conservation across bacteria. ROC curves for individual candidate peptides resulted in AUCs of 0.6-0.85, indicating moderate diagnostic potential within this cohort. However, using the sum of Z scores across multiple peptides, we were able to increase the AUC to 0.96, indicating that by using multiple antigens we can improve sensitivity (**Figure 3B**). Future studies are needed to evaluate performance in other cohorts.

Notably, the most promising diagnostic peptides are from proteins not previously known to be immunogenic. For example, only 19 peptides had a PPV ≥0.9 and sensitivity ≥0.2. All of these peptides are from unique protein clusters and, to our knowledge, none of these proteins have previously been reported as antibody targets. However, in practice, we expect the diagnostic potential of these novel immunogens to vary considerably, due to differences in the level of protein conservation across 1) different strains of *B. pseudomallei* (which can impact sensitivity) and 2) other *Burkholderia* species (which can impact specificity). For example, of 65 proteins of interest screened across predicted proteomes, only 24 (~37%) were conserved in >80% of the screened *B. pseudomallei* strains (n=313) (**Supplementary Table 2**). Additionally, 9/65 proteins (~14%) were conserved in greater than 96% of all screened proteomes and were present in at least one proteome for every screened *Burkholderia* species. To address the issue of sensitivity across strains, we also generated ROC curves using the subset of peptides designed from proteins that were conserved in >80% of screened *B. pseudomallei* proteomes; with this subset, we obtained an AUC of 0.94 (**Figure 3B**).

For each of the 21 peptides selected for having the greatest combined diagnostic potential (using forward and/or backward selection criteria; colored lines in **Figure 3B**) we observed a subset of melioidosis samples with Z scores that were much higher compared to our negative control cohort (**Figure 3C**). One of these selected peptides was designed from the known antigen, GroEL1, whereas the rest, to our knowledge, are from novel antigens. This GroEL1 peptide was designed from near the N terminus (AA positions 22-52) (**Figure 3D**, top). Interestingly, we also observed reactivity against the homologous GroEL2 peptide in a smaller number of individuals (**Figure 3D**, middle). We observed a strong correlation between the Z scores measured for these homologous GroEL1/2 peptides (Pearson R = 0.8), consistent with GroEL1/2 cross-reactive antibodies targeting this epitope. Although only one GroEL1 peptide was selected for inclusion in our diagnostic subset, we also observed reactivity against two additional GroEL1 epitopes (**Figure 3D**), in the absence of reactivity against the homologous GroEL2 peptides. Taken together, this suggests that GroEL1 is the more immunogenic ortholog. One peptide that had particularly high Z scores in ~10% of melioidosis cases in our cohort is annotated as a Toprim domain containing protein (GenBank: WP_236849277.1) (**Figure 3C**). The reactivity against this novel protein antigen was limited to a single epitope near the C terminus (AA positions 220-250) (**Figure 3D**, bottom). Notably, for all three of these proteins (GroEL1, GroEL2 and the Toprim domain containing protein), we observed low levels of reactivity in 1–2 negative control samples, likely indicating some non-specific reactivity at these epitopes (**Figure 3D**).

### 3.2 Peptide tiling with PepSeq to identify mAb epitopes

**Figure 4:**
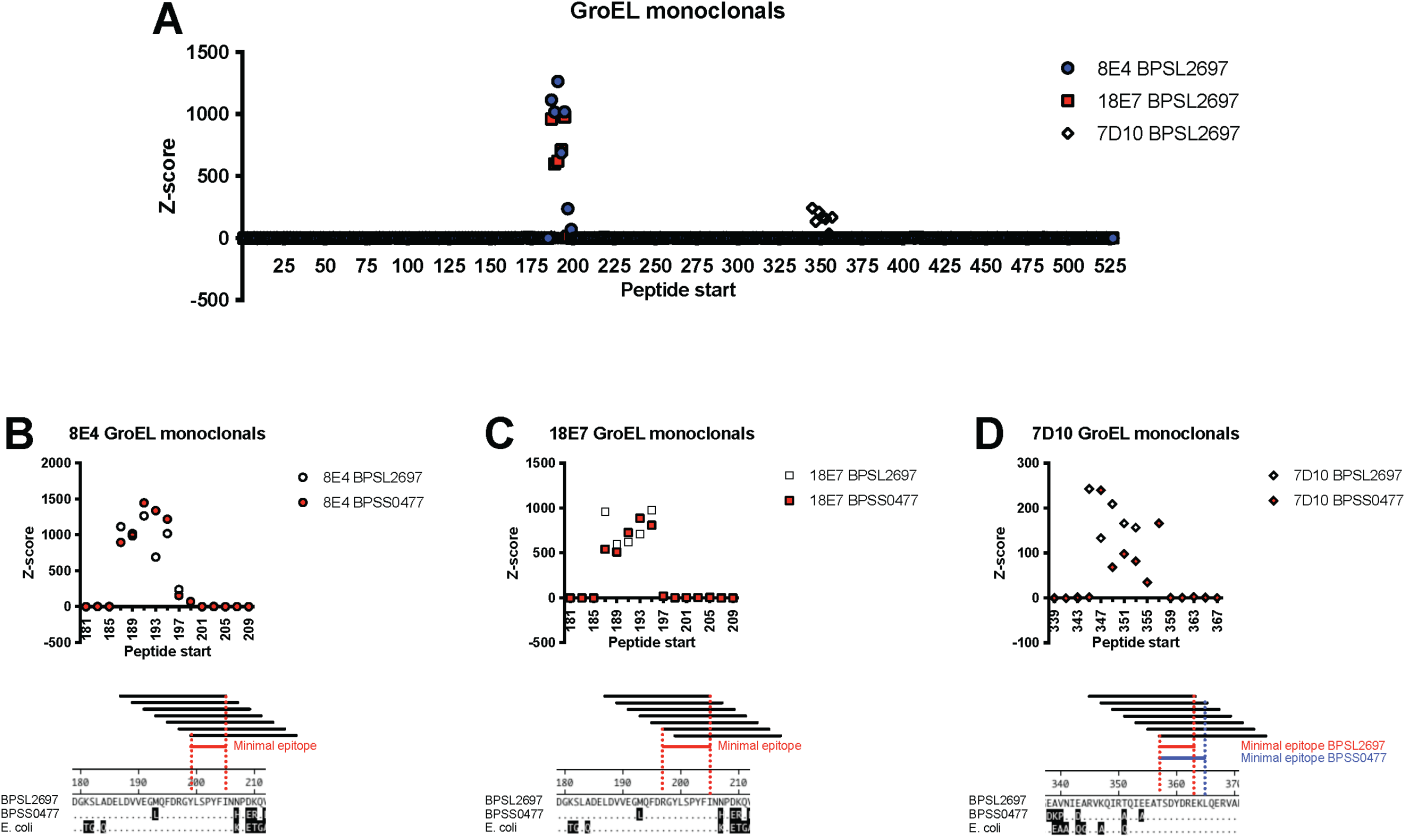
High resolution epitope determination by tiling peptides. Enrichment Z scores for peptides tiled across the GroEL protein highlight regions targeted by three different mAbs. Each peptide is 19 aa and adjacent peptides overlap by 17 aa. **(A)** Antibody binding across the entire GroEL1 protein (BPSL2697) with the different shapes/colors indicating different mAbs. **(B–D)** Subsets of the GroEL protein highlighting the targeted region for each mAb. Overlapping peptides from the homologous GroEL1 (white symbols) and GroEL2 (BPSS0477, red symbols) are shown. The minimal and optimal antibody epitopes are shown for 8E4, 18E7, and 7D10 respectfully. The conservation of these epitopes in the two *B pseudomallei* homologs and *E coli* GroEL is shown in the amino acid alignments. Amino acid differences from BPSL2697 are shown by shaded amino acids.

Six mAbs against GroEL that had been produced for diagnostic assays were tested for their ability to bind to peptides present in the BKP2 PepSeq library, which contains high-density tiled peptides across a subset of *B. pseudomallei* proteins. We found that three of the six mAbs bound to GroEL-derived peptides in a manner consistent with the recognition of a linear epitope. More specifically, we observed strong enrichment (Z score > 10) for each of these mAbs in 6–7 consecutive peptides designed from both GroEL 1 and 2 (**Figure 4A**; average Z score = 115-871). Two mAbs (8E4 and 18E7) bound ligands generated from the same regions of the protein sequence, near and flanking amino acid 190. The third mAb (7D10) bound ligands generated from GroEL sequences near and flanking amino acid 351. Ligands generated from both GroEL 1 and 2 homolog protein sequences (BPSL2697 and BPSS0477) were present in the BKP1 library and these three mAbs showed similar binding profiles to both. Three of the six anti-GroEL mAbs tested did not bind significantly to GroEL peptides, perhaps because they recognize conformational epitopes that are not well represented by 19 aa long peptides.

High resolution definition of each epitope was possible due to the design strategy for BKP2, which included peptides starting every other amino acid across the entire GroEL protein. For mAb 8E4, **Figure 4B** shows 7 tiled peptide sequences and their binding signal. It is possible to discern minimal peptide sequences based upon the binding signals of individual ligands (i.e., AA sequence shared across all peptides with average Z score ≥ 10). The 18E7 mAb is distinct and independently generated from 8E4 yet shows a very similar binding pattern. The third mAb, 7D10, bound peptides from a different protein region and its epitope is distinct. In both regions, the amino acid sequences defining the epitopes are highly conserved across the *B. pseudomallei* homologs and the *E. coli* GroEL. While not directly tested, these three mAbs would not be expected to differentiate amongst these antigenic proteins.

### 3.3 PepSeq design strategies to finely characterize antibody epitopes

The flexibility of the PepSeq library design and construction processes allowed us to further explore the binding of these mAbs at the identified epitopes. To do this, we assessed the binding profiles of the 8E4 and 7D10 mAbs using a bespoke PepSeq library (MUT) that contained 30 aa “parent” peptides designed from GroEL1 and centered on each identified epitope (see section 3.2), along with 620 variants of each of these parents. By comparing levels of reactivity across parent and variant peptides, we were able to finely dissect interactions between the mAbs and their cognate antigens.

**Figure 5:**
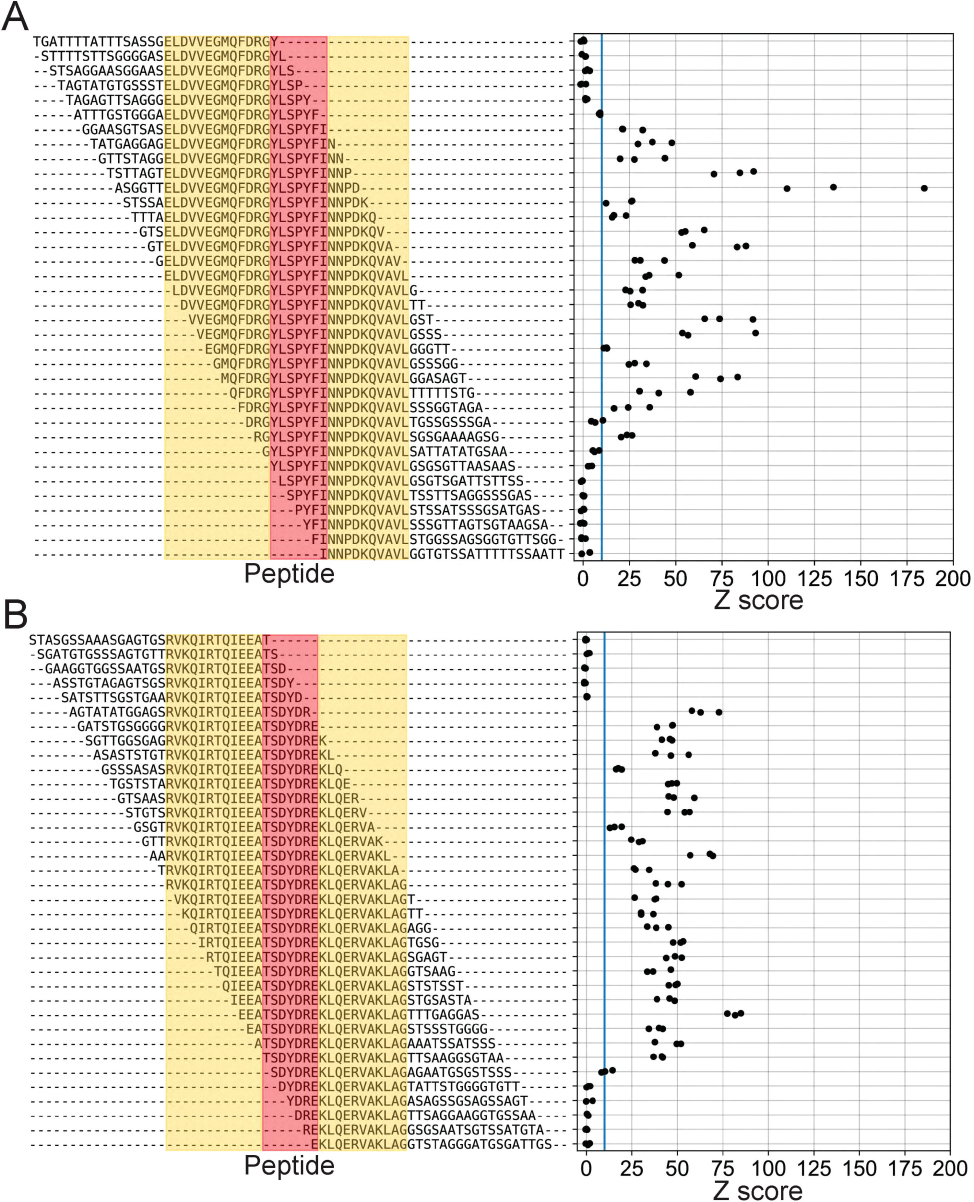
High-resolution scanning finely maps span of linear epitopes. Enrichment Z scores from assays of mAbs 8E4 **(A)** and 7D10 **(B)** using the MUT PepSeq library. Each row represents a single peptide contained in the MUT PepSeq library. Red is used to highlight the previously identified “minimal” epitopes (**Figure 4**), while yellow highlights additional GroEL-derived residues. Residues that are not highlighted represent randomly generated combinations of alanine, glycine, serine and threonine (i.e., sequences not present in the GroEL protein). Blue vertical lines are included in each panel to indicate Z = 10. Each black circle represents a single PepSeq replicate; three replicates were run for each mAb.

First, we wanted to finely map the minimum binding footprint of these mAbs by independently testing different subsets of the parent peptide in isolation. To do this without modifying the overall peptide length, we added randomly generated sequences to replace the portions that had been removed (**Figure 5**, left). As expected, we observed strong enrichment with each mAb for several variant peptides containing the previously defined epitopes (**Figure 5**). However, we observed some small differences in the minimal epitopes identified by the BKP2 and MUT libraries (8E4: BKP2 = YLSPYFI, MUT=RGYLSPYFI; 7D10: BKP2 = TSDYDRE, MUT=SDYDR; shared sequence regions are underlined). This is likely due to the difference in tiling density (step size of 2 vs 1), peptide length (19 vs 30 aa) and/or differences in the sequence flanking the epitope. Comparing across these “shift” variants also illustrated how small changes in the sequence context of an antibody epitope can have a substantial impact on the magnitude of PepSeq enrichment. There are several pairs of very similar peptides, both of which contain the mapped antibody epitope, which result in substantially different enrichment Z scores. For example, for 8E4, the average Z score, across replicates, for ASGGTTELDVVEGMQFDRGYLSPYFINNPD was 143.3, while the average Z score for STSSAELDVVEGMQFDRGYLSPYFINNPDK was 21.6 (shared sequence regions are underlined). This may be related to changes in secondary structure that modify the way the epitope is presented.

**Figure 6:**
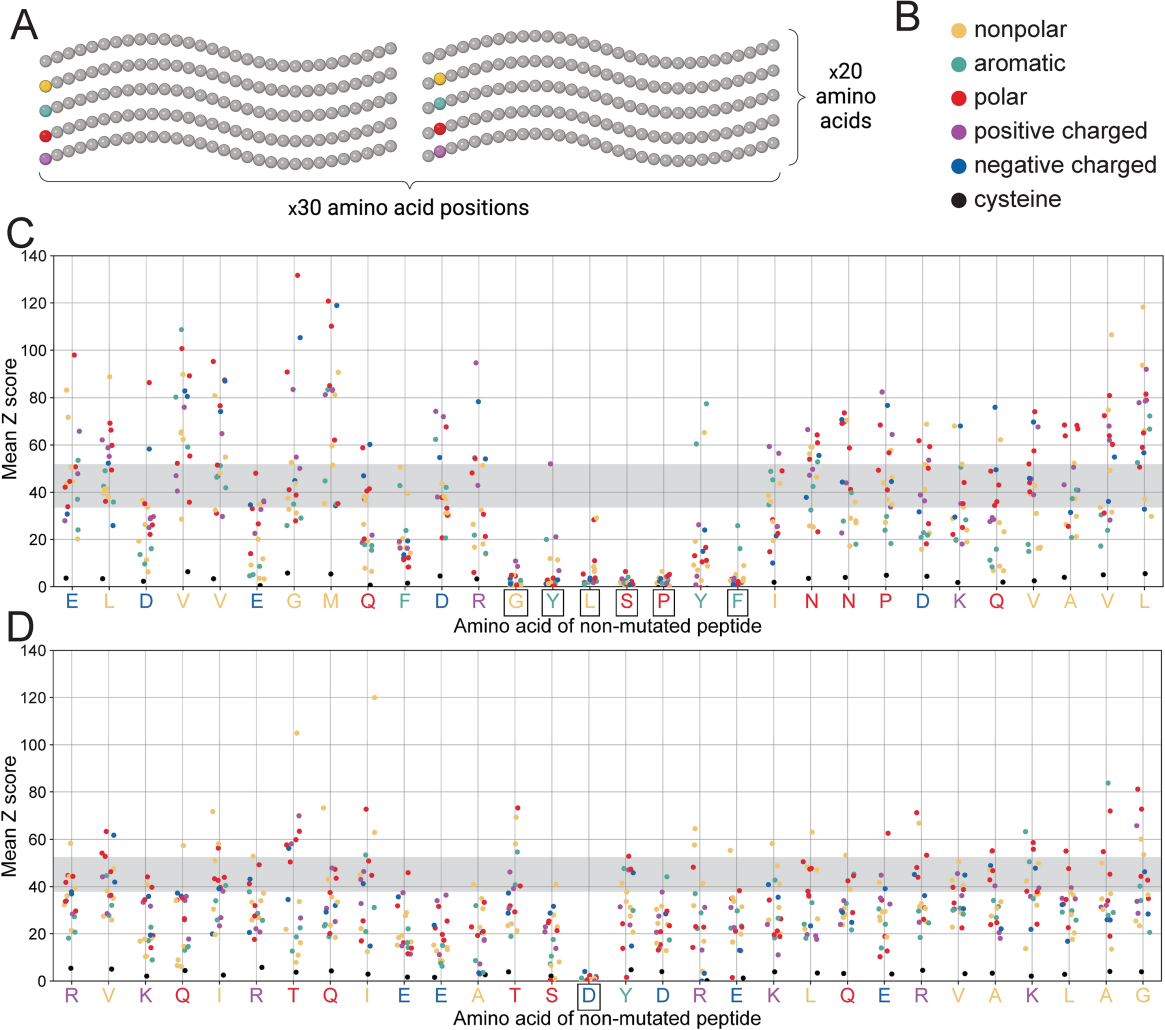
PepSeq-based saturation mutagenesis highlights critical residues for antibody binding. **(A)** A diagram depicting our saturation mutagenesis strategy, as described in section 2.5. Created with BioRender.com. **(B)** Legend for colors used in panels C-D. Different colors are used for different amino acids, based on their biochemical properties. **(C, D)** contain scatterplots depicting PepSeq reactivity for each of the 570 saturation mutagenesis variants designed for the 8E4 **(C)** and 7D10 **(D)** mAbs. The x-axis indicates the wild-type peptide and the positions of the mutated residues (with a random jitter added to aid with visualization), while the y-axis indicates the average Z score across three replicates. Individual points are colored as indicated in **(B)**. The horizontal gray bands indicate the range of Z score values for the non-mutated, wild-type peptide. Boxes around the wild-type amino acids (x-axis tick labels) indicate residues that are individually critical for antibody binding, meaning the mean Z score for ≥12/19 amino acid substitutions at these positions was <5.

Second, we wanted to better understand the importance of individual residues within these antibody epitopes. We did this by designing a set of variants in which each residue in the parent peptide was individually substituted for each of the 19 non-wild-type amino acids (30 positions x 19 amino acids = 570 variants per mAb) (**Figure 6A**). The parent amino acid sequences are shown in **Figures 6C, D** along the X axis and match the *B. pseudomallei* GroEL1 protein exactly. By comparing binding levels across these epitope variants, we were able to identify individual, critical residues within each epitope (i.e., positions where most amino acid substitutions eliminate antibody binding; boxed residues in **Figure 6**). These data reveal the full spectrum of amino acid substitutions that can occur without impacting antibody binding, which can help us to understand the potential for *B. pseudomallei* to evade recognition through evolution.

For both of these mAbs, the majority of the parent peptide residues were associated with a broad range of Z scores, when substituted to each of the 19 non-wild-type amino acids (**Figures 6C, D**). Although most of these residues fall outside of the optimal antibody epitopes, their substitution may still impact antibody binding (both positively and negatively), for example, by modifying the secondary structure of the PepSeq probe. However, with one exception, we did not find that a particular amino acid was consistently associated with increases or decreases in reactivity (**Supplementary Figure 4**). The one exception was cysteine. Substitution to cysteine consistently decreased Z scores, regardless of which residue position was mutated (**Figure 6**, **Supplementary Figure 4**). This is likely related to the unique ability of cysteine to facilitate the formation of disulfide bonds.

However, for both mAbs there were a subset of positions at which an amino acid substitution almost universally abrogated binding, regardless of the nature of the amino acid substitution (**Figures 6C, D**), and all these positions were located within the minimal epitopes described above. Presumably, these are sites that play a critical role in facilitating the binding of antibody and antigen, in a manner that cannot be compensated by other, neighboring interactions. Therefore, mutations at these positions could allow for escape from these mAbs, assuming that they do not interfere with normal protein function. Notably, we only observed a single critical residue in the 7D10 epitope, aspartic acid at position 15 (**Figure 6D**). In contrast, for the 8E4 mAb we observed six individually critical epitope residues (**Figure 6C**). Therefore, there are more potential escape pathways for the 8E4 mAb compared to the 7D10 mAb.

**Figure 7:**
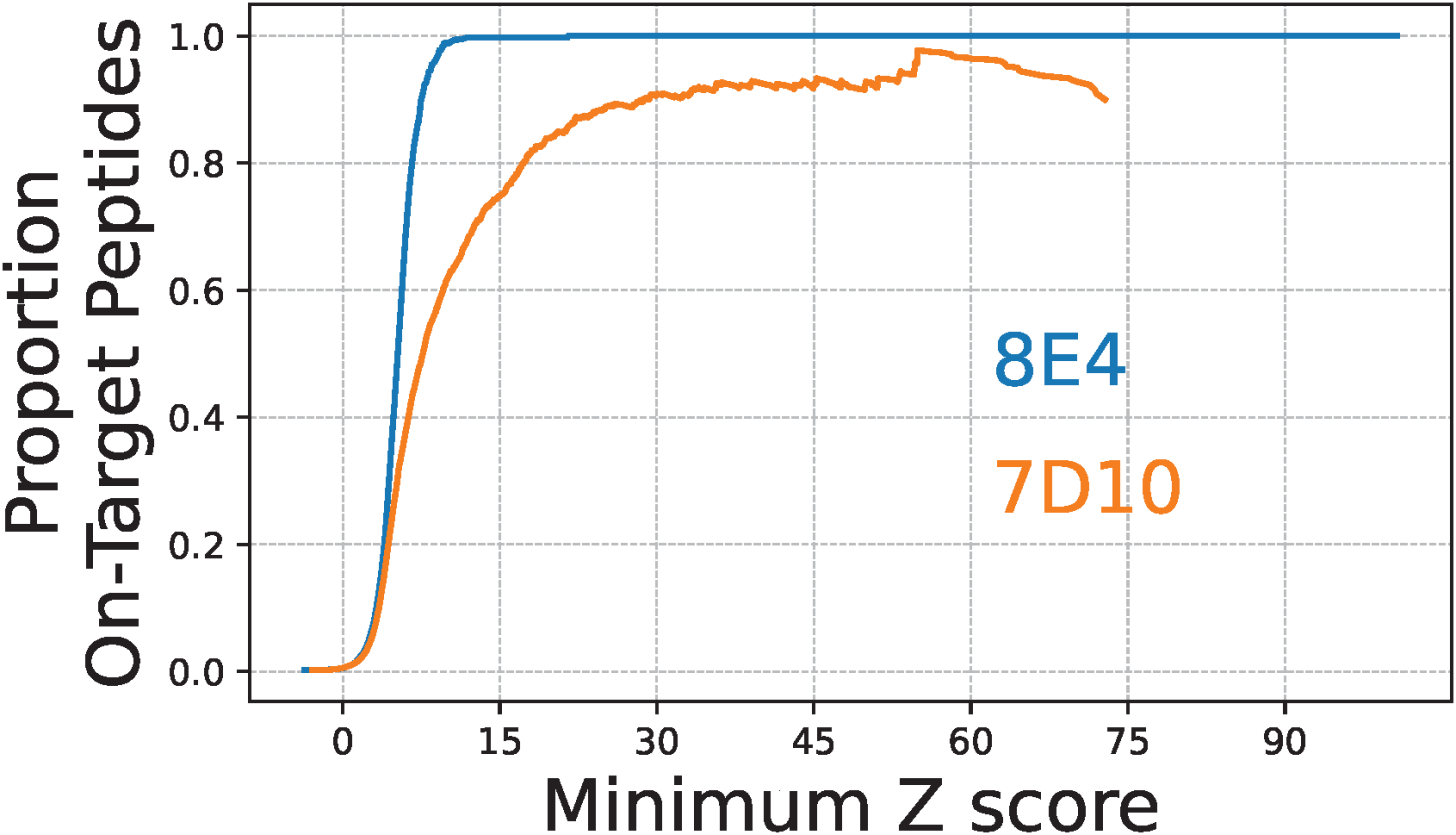
The 8E4 and 7D10 monoclonal antibodies differ substantially in specificity for their cognate GroEL antigens. Lines indicate the proportion of on-target peptides with average PepSeq Z scores greater than or equal to a range of Z score thresholds: blue = 8E4, orange = 7D10. All Z score thresholds are inclusive of ≥10 peptides.

Finally, we evaluated the specificity of the 8E4 and 7D10 mAbs by putting our on-target peptide Z scores in the broader context of those measured across the full MUT library (**Figure 7**). For each mAb, the entire set of on-target peptides represented just 0.25% of the full library. This analysis indicated that the 8E4 mAb is highly-specific to its cognate antigen, at least in terms of the diversity covered by this PepSeq library. More than 99.75% of the MUT peptides with average 8E4 Z scores ≥15 were designed from the relevant GroEL epitope. In contrast, 7D10 bound strongly to a variety of off-target peptides, in addition to its cognate GroEL epitope. Only ~75% of the peptides with average 7D10 Z scores ≥15 were designed from the relevant GroEL epitope. Setting an average Z score threshold of 15, we observed 162 off-target peptides enriched in our assays with 7D10. These off-target enriched peptides were representative of 26 non-*Burkholderia* epitopes, which is significantly fewer than expected with random sampling from the off-target peptides contained in the MUT PepSeq library (p<1e-6; from 1,000,000 random simulations, the smallest number of epitopes represented in a given set of 162 off-target peptides was 113). Taken together, we conclude that the 7D10 mAb binds to a motif that is commonly observed across a wide variety of proteins.

### 3.4 Surface plasmon resonance studies to estimate binding affinities

Peptide epitopes could have different binding characteristics compared to the full-length protein, even though the epitope would be linear in both situations. Any changes in this interaction could influence the potential downstream diagnostic assays that only use peptides. To understand how the mAbs bind to shorter peptide epitopes relative to the full-length native proteins, SPR studies using two mAbs were conducted with both types of ligands. In **Table 1**, data are presented where full length, recombinant GroEL (rGroEL) and the centered optimal 19 amino acid peptide for 8E4 and 18E7 (13004-bio - EGMQFDRGYLSPYFINNPD) were attached to the SPR chip. Both of these antibodies were used since they recognize the same epitope region (**Figure 4**). 7D10 binding was only measured against rGroEL. The rGroEL was attached directly while 13004-bio was bound via an N-terminus biotin which subsequently was bound to streptavidin attached to the surface. SPR provided kinetic rate constants for k_a_ (association rate), k_d_ (dissociation rate), and then the steady state K_D_ for each binding interaction. Because identification of 13044-bio was based upon mAb binding, the SPR confirmation of binding was expected. For both 8E4 and 18E7, the K_D_ was similar between 13004-bio and rGroEL (82–144 nM), though the k_d_ rates were faster for the shorter peptide. In all cases, 8E4 had higher affinity than 18E7. Experiments where the mAb was attached to the surface and rGroEL and 13004-bio were the free ligands were consistent with these results (data not shown). In combination, the faster disassociation rate of peptides could have negative implications when using peptides in diagnostic assays.

**Table 1:** mAb binding affinity for GroEL protein versus epitope-defined peptide.

Analyte	Immobilized Ligand	Steady State K_D_ (nM)	k_a_ (1/Ms)	k_d_ (1/s)
8E4 mAb	GroEL peptide	106	5.04x10^4^	5.85x10^-3^
	rGroEL	82	4.56x10^4^	6.61x10^-4^
18E7 mAb	GroEL peptide	118	3.44x10^4^	1.43x10^-2^
	rGroEL	144	8.70x10^4^	4.98x10^-5^
7D10 mAb	GroEL peptide	ND	ND	ND
	rGroEL	56	5.72x10^4^	2.92x10^-3^

Surface Plasmon Resonance was used to measure mAb binding rate constants for full length recombinant GroEL (rGroEL) and a 19 amino acid GroEL peptide identified within the GroEL protein (see **Figure 4**). These data were generated with rGroEL and GroEL peptide attached to the surface and the mAb free to bind, but when this was reversed (mAbs attached) similar rate constants were obtained (data not shown). ND, Not determined.

## 4 Discussion

Antibodies are a key component of the adaptive immune response and often serve as critical mediators and correlates of immunity following vaccination. However, the antibody response is also vastly diverse. For example, the naïve antibody repertoire in humans is estimated to include at least 10^12^ unique proteins (27), and even more diversity can be generated through somatic hypermutation. Collectively these antibodies target a wide variety of antigens with differing specificities and affinities, both of which have important implications for function and therefore protection from future infections. Traditional approaches for characterizing antibody responses barely scratch the surface of this complexity and therefore provide an incomplete view of the antibody responses stimulated by vaccines and natural infections. Highly multiplexed serological approaches, like PepSeq, are allowing us to obtain a more complete view of this complexity, with implications for vaccine design, evaluation and beyond. Here, we demonstrate several applications of the PepSeq platform for highly multiplexed serology using an important biodefense pathogen, *B. pseudomallei*: 1) identification of novel antigens using a proteome-wide peptide library and 2) fine-scale characterization of antibody epitopes using high resolution peptide tiling in combination with bespoke libraries of peptide variants.

Antigen discovery is a critical, early step in the development of modern vaccines and can also aid in the design of serological diagnostics. Modern, directed subunit vaccines focus on the delivery of a small number of immunodominant targets, known to stimulate protective immune responses. However, the selection of vaccine antigens is often non-trivial, especially for complex pathogens, like bacteria, fungi and eukaryotic parasites, which have large, and often highly variable, proteomes (28). Although they cannot directly measure level of protection, highly multiplexed serology assays are enabling the identification of antigenic proteins at an unprecedented scale. A single assay can measure antibody binding against 1000s of protein targets using <1 μl of serum/plasma as input, and new assays can be built, from scratch, in a matter of weeks, regardless of the pathogen/targets of interest. By identifying proteins commonly targeted by antibody responses to natural infections, or in response to complex vaccines (e.g., live-attenuated or inactivated), we can generate a short list of candidate antigens that can be prioritized for future investigations.

As a proof-of-concept for the application of PepSeq to a complex pathogen, we designed several libraries to study the antibody response to *B. pseudomallei*. For a bacterial pathogen, *B. pseudomallei* has a relatively large genome, typically ~7–8 Mb in size. Individual strains of *B. pseudomallei* generally encode >6000 protein coding genes, and gene content is highly variable across strains (28). Therefore, to fully cover the species-wide antigenic proteome space of this bacterium, we needed to assess antibody responses against >10,000 proteins, which would not be feasible using traditional approaches for serology, or even more contemporary, moderately multiplexed methods, such as the Luminex platform (29) and NAPPA microarrays (30). Using a PepSeq library containing ~244,000 *B. pseudomallei*-derived peptides, we identified 65 proteins as priorities for further investigation. These are proteins that elicited robust antibody responses that were largely specific to individuals with documented *B. pseudomallei* infections, who are likely to be protected against future infections (31). Therefore, they represent good candidates for use in vaccines and diagnostics. To further understand the diagnostic potential of these antigens, it will be important to profile additional cohorts, including melioidosis confirmed cases from other endemic locations, as well as negative controls that are well-matched for potentially confounding variables, such as geography and co-morbidities like diabetes.

It is also important to note that the set of proteins identified in this study is likely a subset of those targeted by antibodies in response to melioidosis. Highly multiplexed approaches to serology utilize peptides as antigens and therefore are not expected to detect antibodies that bind specifically to conformational epitopes formed through tertiary and quaternary structures (7). Although many immunogenic proteins are likely to contain both linear and conformational epitopes, our sensitivity will vary depending on the relative abundance of these two epitope types. Therefore, serological approaches using more complex antigens (e.g., protein arrays) should provide complementary data and will help to further characterize the antibody responses stimulated by complex pathogens like *B. pseudomallei*.

One notable finding of our study is that there is a high level of variability, among individuals with confirmed melioidosis, in terms of which proteins are targeted by the antibody response. This is reflected in the modest levels of sensitivity provided by individual peptides (**Figure 3A**) along with the large number of proteins targeted by antibodies in at least one individual. This variability in the antibody responses to natural infections is not necessarily problematic for vaccine development. By delivering a subset of antigens, modern vaccines can direct the immune response to specific immunogens. However, to be good candidates as vaccine antigens, these proteins must have the capacity to stimulate protective antibodies; therefore, future work is needed to characterize the functionality of antibodies directed against our candidate antigens.

The observed variability in targeted *B. pseudomallei* antigens is more problematic from a diagnostic perspective; however, we do not view this as an insurmountable barrier. We have already shown how sensitivity can be increased by using a combination of peptides/proteins (**Figure 3B**). It may also be possible to increase sensitivity by transitioning our candidates to other serology platforms that can utilize larger, more complex antigens (e.g., full proteins or domains). This is because highly multiplexed approaches, like PepSeq, utilize peptides as antigens, and therefore, they can only directly measure a subset of the antibody response (i.e., they cannot measure antibodies that require tertiary or quaternary structures for binding). Assuming that immunodominant linear epitopes will be commonly observed within more generally immunodominant proteins (consistent with our identification of dominant epitopes within several known *B. pseudomallei* antigens), we may have only measured a subset of the antibody response against our candidate proteins. In support of using large domains or proteins, we have shown that peptides have a higher disassociation rate compared to the full-length protein (**Table 1**), which may hinder sensitivity. However, transitioning to larger versions of these antigens also comes with the risk of reduced specificity due to cross-reactive antibody responses.

Another important advantage of highly multiplexed serology is the ability to finely characterize antibody epitopes, which can aid in the design of antigens for use in vaccines and diagnostics, as well as inform on the potential for pathogen escape from immune responses and antibody-based therapeutics. Near epitope-level resolution is inherent to the data generated by platforms like PepSeq because of the use of relatively short, peptide antigens. However, we have also shown that, thanks to the highly multiplexed nature of these assays and the ability to fully define the peptides contained in an assay, it is possible to understand antibody binding footprints down to individual residues. To demonstrate this potential, we characterized a panel of mAbs that were previously developed against the *B. pseudomallei* GroEL1 protein, which is known to be a strong antigen during melioidosis. First, we used a high-density, tiled peptide design to determine which mAbs recognized linear epitopes and to determine the location of the targeted epitope within the GroEL protein. Out of a panel of six mAbs, 50% bound strongly to GroEL peptides in our assay, and we were able to determine that these three mAbs targeted two distinct regions of GroEL that were highly conserved across the two homologs present in the *B. pseudomallei* genome.

Following the initial identification of these epitopes, we then designed a library of peptide variants specific to these regions of GroEL. By quantitatively measuring mAb binding simultaneously across this collection of variants, we were able to finely assess how these antibodies were interacting with their cognate antigens. Not only did this approach allow us to define minimum binding footprints, but we were also able to assess the impact of individual amino acid substitutions, and we observed strikingly different impacts at our two mAb epitopes. Although both of our epitopes could be mapped to a minimal region of ~5–9 amino acids within the GroEL protein, for one (bound by 8E4), most of the residues contained in this region were individually critical, meaning that most individual substitutions at these positions resulted in a complete loss of antibody binding in our assay. Whereas, for the second epitope (bound by 7D10), only a single residue was individually critical, suggesting that there are redundant interactions that can maintain affinity even in the face of evolution. This characteristic should make 7D10 more resistant to immune escape, but it also appears to have impacted the specificity of the mAb. By simultaneously measuring binding to peptides from many distinct epitopes (designed from different viruses and bacteria), we demonstrated that 7D10 cross-recognizes many other known B cell epitopes, while 8E4 is much more specific to GroEL. Therefore, our characterization of these two anti-GroEL mAbs illustrates the way that fine-scale characterization of binding profiles can inform the downstream utility of antibodies as therapeutics.

## Data Availability

The original contributions presented in the study are included in the article/**Supplementary Material**. Further inquiries can be directed to the corresponding author/s.
