# Supplementary material for: PepSeq as a highly multiplexed platform for melioidosis antigen discovery and vaccine development
Source: Front Immunol. 2025 Jul 3;16:1605758. doi: 10.3389/fimmu.2025.1605758 (PMC12267161; doi:10.3389/fimmu.2025.1605758)
Supplement: Supplementary file 1 [file DataSheet1.pdf]

Supplementary Methods

BKP1 Library Design

For our full "pan proteome" PepSeq library design (BKP1), we downloaded all protein sequences linked to txid28450 in GenBank (RefSeq) on 2023-02-23 (408,652) and in UniProt (Swiss-Prot and TrEMBL) on 2023-03-02 (122,480 sequences). After removing duplicates (using https://github.com/LadnerLab/Library-Design/tree/master/one_hundred_reps/python) and sequences < 30 aa in length, we were left with 346,363 target amino acid sequences. We used uclust (1) to group together similar sequences, initially using a threshold of 50% amino acid identity, which resulted in the formation of 25,500 clusters. We later decided to split 50 of these initial clusters at a higher identity threshold (90%) to ensure good coverage of known *Burkholderia psuedomallei* core genes.

To focus our design on proteins that are more commonly present among different isolates and/or more heavily studied, we utilized the subset of clusters with ≥3 representative proteins for peptide design. However, we also included ~200 clusters with <3 sequences each because they matched known *B. psuedomallei* core genes. We also excluded 27 clusters (with 3 sequences each) that had annotations including the word "phage".

Low complexity peptides were removed from the final design, as these are likely to be recognized by non-specific antibody responses. Specifically, peptides were removed is they failed to meet one or more of these criteria: 1) ≥7 unique amino acids contained in the full peptide, 2) no consecutive homopolymers ≥8 amino acids in length, 3) most common amino acid composing ≤13 out of the 30 total residues (≤43.33%), and 4) ≤5 copies of any single 3mer motif.

MUT Library Design

The full MUT PepSeq library contains 243,855 target peptides, each of which is 30 amino acids in length. 1242 of these peptides were used to extensively cover two regions of the *B. pseudomallei* GroEL1 protein, which were previously identified (using the BKP2 PepSeq library) as containing mAb epitopes (8E4-18E7: ELDVVEGMQFDRGYLSPYFINNPDKQVAVL, 7D10: RVKQIRTQIEEATSDYDREKLQERVAKLAG; inferred epitope regions are underlined). The other 242,613 peptides were designed for another project and therefore, can be considered here as negative controls. These 242,613 peptides were designed to interrogate a total of 613 additional antibody epitopes contained in a variety of different proteins; 379 epitopes were each represented by 621 peptides (as described for the GroEL epitopes), while 234 epitopes were each represented by 31 peptides.

References Cited

1. Edgar RC. Search and clustering orders of magnitude faster than BLAST. *Bioinformatics* (2010) 26:2460–2461. doi: 10.1093/bioinformatics/btq461
