# Supplementary material for: PepSeq as a highly multiplexed platform for melioidosis antigen discovery and vaccine development
Source: Front Immunol. 2025 Jul 3;16:1605758. doi: 10.3389/fimmu.2025.1605758 (PMC12267161; doi:10.3389/fimmu.2025.1605758)
Supplement: Supplementary file 2 [file DataSheet2.pdf]

Supplementary Figures

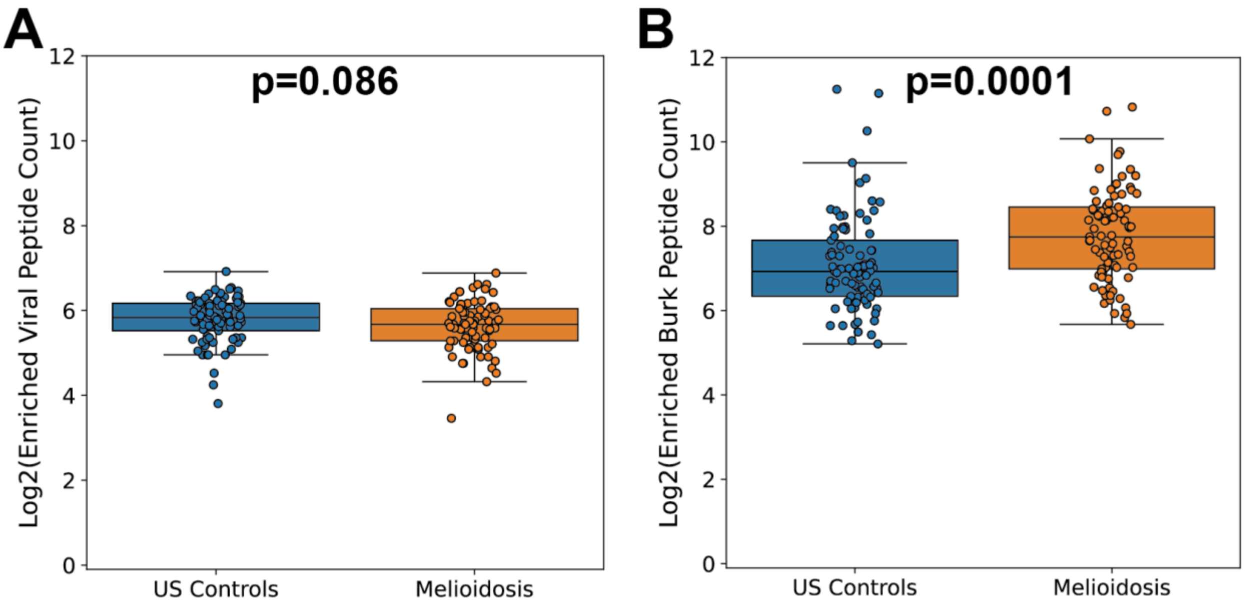

Supplementary Figure 1. Differences in overall number of enriched viral control and B. pseudomallei derived peptides between cohorts. Boxplots showing the counts of enriched peptides for the viral control peptides (A) and *B. pseudomallei* derived peptides (B) for all sera samples (n=174). Box and whisker plots show the median, standard deviation and 95% confidence of each reactivity distribution. P-values from two-tailed independent t-test comparing US Controls and Melioidosis cohorts is indicated at the top of each plot.

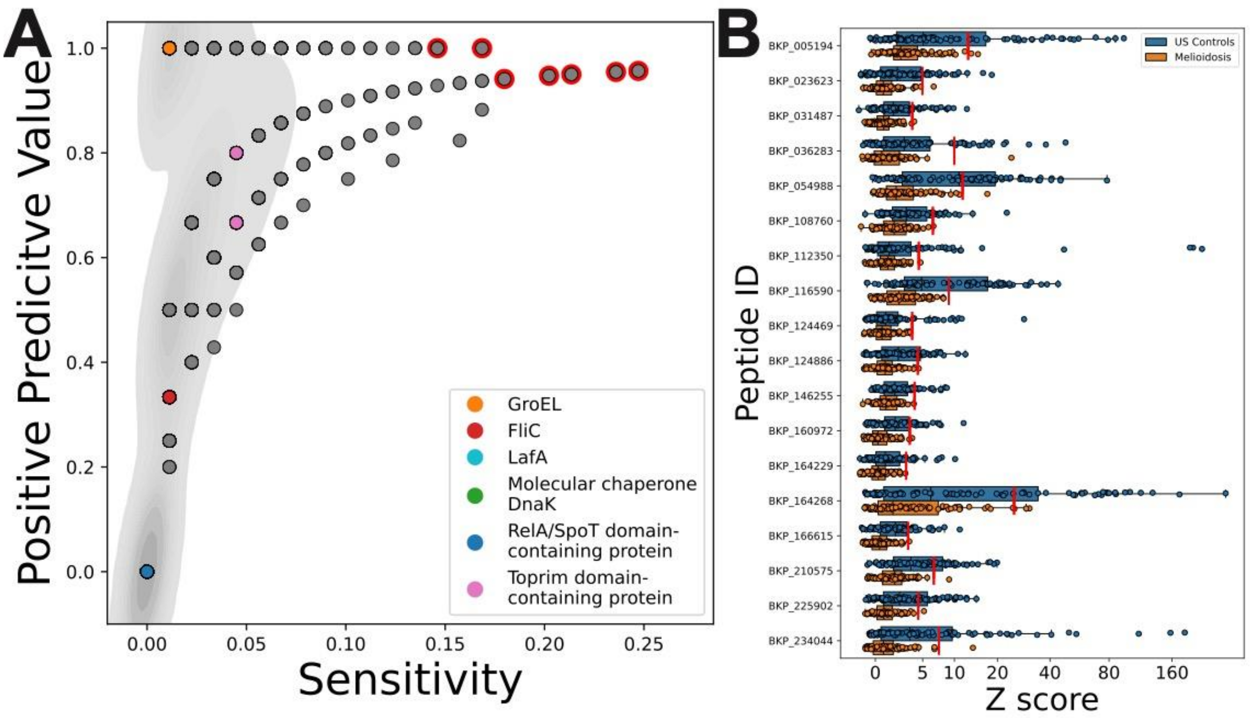

Supplementary Figure 2. Reverse analysis of peptides with increased reactivity in the control cohort compared to the melioidosis cohort. (A) For a subset of 4,435 peptides recognized by serum IgG antibodies in ≥4 samples, the positive predictive value is plotted versus the sensitivity. Red outline indicates peptides that were significantly elevated in control samples (two-sided Fisher's Exact Test). Grey shading in background indicates the density of points. (B) Antibody reactivity for the 18 peptides identified as being significantly elevated in Control serum samples. X-axis indicates Z score of each peptide and the y-axis shows the peptide ID. Each circle represents an individual sample. The line within each box represents the median, while the lower and upper bounds of each box represent the first and third quartiles, respectively. The whiskers extend to points that lie within 1.5 interquartile ranges of the first and third quartiles. Red lines show the Z score threshold calculated for each peptide.

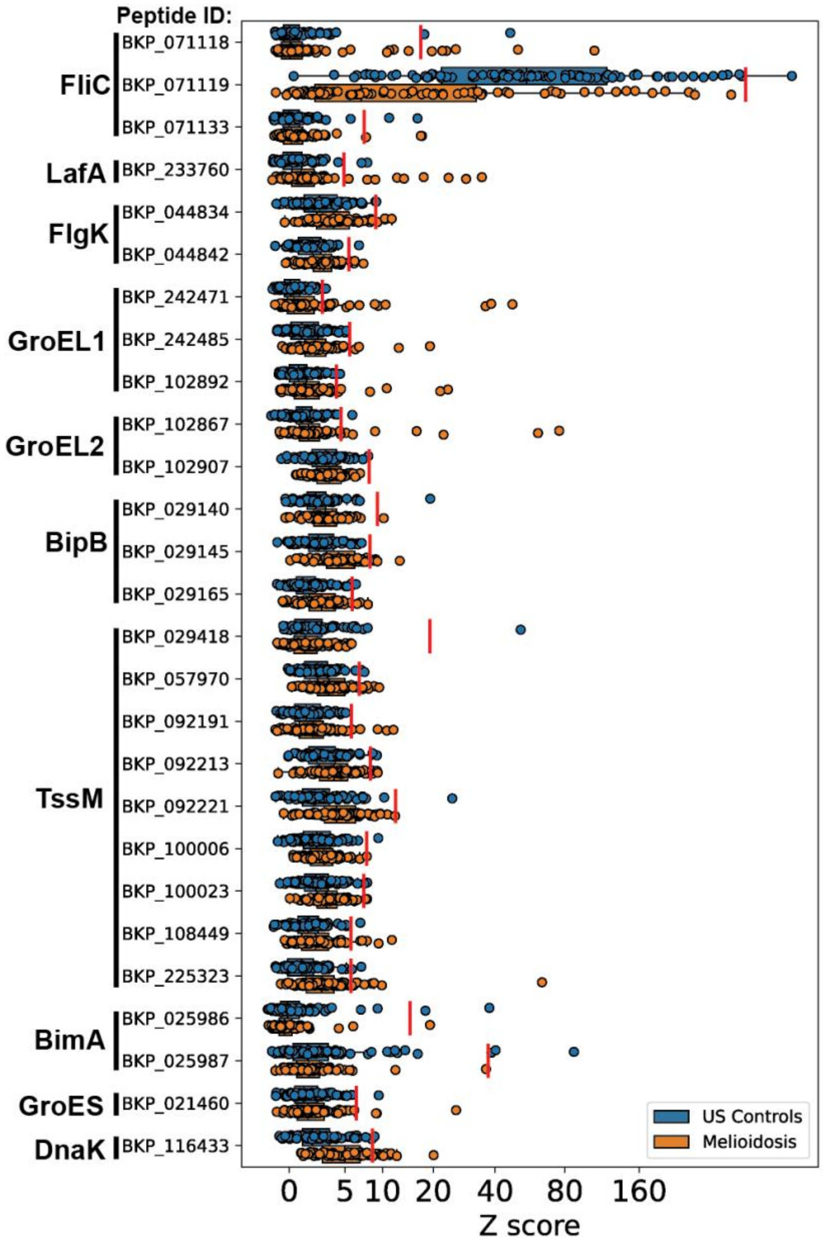

Supplementary Figure 3. Median Z scores for Melioid and Control Sera for peptides designed from known antigens. The median reactivity for peptides that had a Z score > 6 in at least 4 samples and were designed from proteins that have been previously identified as being antigenic is shown for each sera specimen (N=174). The protein abbreviations indicating which protein each peptide was designed from are shown on the far left. Detailed information about each peptide can be found in Supplemental Table 1. Box and whisker plots show the median, standard deviation and 95% confidence of each reactivity distribution. Red lines show the Z score threshold calculated for each peptide.

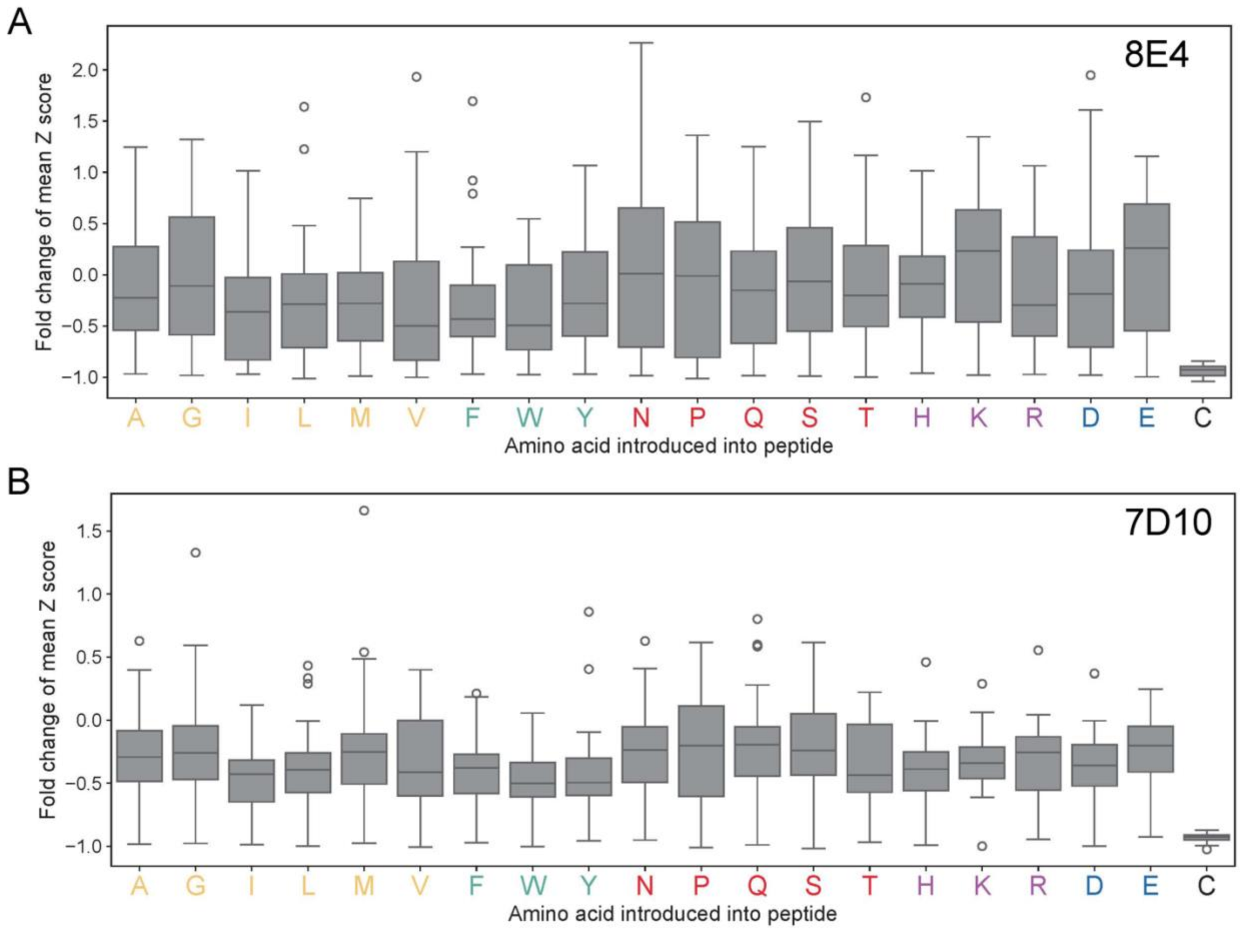

Supplementary Figure 4. Mean fold change of Z scores for each amino acid introduced into mutated peptides. Boxplots showing the distribution of mean fold changes for mutated peptides compared to the original peptide for each amino acid introduced into 570 saturation mutagenesis variants designed for the 8E4 (A) and 7D10 (B) mAbs. The x-axis indicates the introduced amino acid into a mutated peptide. The y-axis indicates the mean fold change in Z score of a mutated peptide compared to the original peptide. Mean fold change was calculated with (mean Z score of mutated peptide – mean Z score of original peptide) / mean Z score of original peptide.
